# THE MULTIDIMENSIONAL SUCCESSFUL AGING SCALE: DEVELOPMENT AND VALIDATION

**DOI:** 10.1093/geroni/igac059.985

**Published:** 2022-12-20

**Authors:** Edwin K H Chung, Dannii Yeung

**Affiliations:** City University of Hong Kong, Kowloon, Hong Kong; City University of Hong Kong, Kowloon, Hong Kong

## Abstract

Subjective successful aging involves a self-appraisal of individual aging process over multiple dimensions of later life (Feng et al., 2015). However, studies often ignore its multidimensional nature and simply utilize a single item as the measurement (e.g., Stewart et al., 2019). Two studies were hence conducted to develop and validate the Multidimensional Successful Aging Scale (MSAS). In Study 1, MSAS was administered among 414 community-dwelling older Chinese adults (Mage=64.50 years, SD=4.01; range = 60 – 82; 55.3% females). Repeated exploratory factor analysis revealed a nine-factor solution of 25 items, accounting for 64% of the variance. The nine factors include harmonious family, active engagement, supportive friendship, adaptive coping, social contribution, living independently, civic awareness, positive attitudes, and perceived constraints. The nine factors exhibit similar strength of associations with most of the well-being measures, but certain factors show stronger correlation with depressive symptoms, suggesting the uniqueness of each factor. In Study 2, the nine-factor model of MSAS was replicated in a sample of 558 older adults (Mage=69.11 years, SD=6.68; range = 60 – 97; 59.2% females) and the results of CFA indicated a good model fit (χ2 = 527.86, df = 239; CFI =.97; TLI =.96; RMSEA =.05; SRMR = .04). The strict and metric forms of measurement invariance were shown across age groups and gender, respectively. Overall, the MSAS demonstrates satisfactory psychometric properties. These findings disclose that the older adults’ perceptions of successful aging cover broader dimensions than those identified in the Rowe and Kahn’s (1997) model.

